# Elevating the Impact of Nutrition and Obesity Policy Research and Evaluation

**DOI:** 10.5888/pcd12.150142

**Published:** 2015-04-30

**Authors:** Sheila Fleischhacker, Jennifer J. Otten, Elizabeth A. Dodson, Sameer Siddiqi

**Affiliations:** Author Affiliations: Jennifer J. Otten, University of Washington School of Public Health, Seattle, Washington; Elizabeth A. Dodson, Brown School and Prevention Research Center in St Louis and Washington University in St Louis, St Louis, Missouri; Sameer Siddiqi, Johns Hopkins University Bloomberg School of Public Health, Baltimore, Maryland. At the time this essay was conceived, Mr. Siddiqi was with the Applied Research Program, Division of Cancer Control and Population Sciences, National Cancer Institute, Bethesda, Maryland.

Increasingly, public policy is recognized as a high-impact and robust approach for accelerating progress toward reducing and managing nutrition-related chronic diseases such as obesity (1). In various jurisdictions, policy makers enact courses of action, regulatory measures, laws, and policies and set funding priorities designed to improve access to healthier food and beverage options (2). Public policy, however, is often the least understood strategy for creating supportive nutrition environments for population health impact. Research has predominantly focused on understanding individual behavior change rather than evaluating approaches to environmental, policy, and system-level change (1,3,4). More attention has been given recently to approaches that could potentially strengthen our understanding of policy including empirical public health law and policy; research, dissemination, and implementation of science; and public health policy evaluation and research (5). Nevertheless, little is known about whether or how nutrition and obesity policy research and evaluation findings influence policy pathways or whether these findings are consistently and systematically used in formulating public policy.

To explore the evidence as well as promising practices in this area, the Nutrition and Obesity Policy Research and Evaluation Network (NOPREN) Policy Research Impact Working Group (PRIWG) formed in 2011. NOPREN is a thematic Prevention Research Center network created in 2009 by the Centers for Disease Control and Prevention (CDC), Division of Nutrition, Physical Activity, and Obesity, to conduct transdisciplinary nutrition and obesity-related policy research and evaluation across a policy continuum (4). NOPREN participants leverage expertise, funding, and resources across the network and have led the formation of working groups to help coordinate and enhance efforts in areas of common interest and need. We reflect here on the process and potential of PRIWG to improve understanding about and build connections between researchers and policy makers and to explore how to better use these connections in conducting and communicating nutrition and obesity policy research, from initial idea generation through findings dissemination.

Two NOPREN members (J.J.O. and E.A.D.) created PRIWG and recruited fellow NOPREN participants. One of the first PRIWG undertakings is published in this issue of *Preventing Chronic Disease* (6). Briefly, to enhance understanding about the state of public health researcher practices and beliefs and the barriers and facilitators to communicating and engaging with policy makers, 18 semi-structured interviews were conducted with public health nutrition and obesity researchers who are highly involved in communicating research to policy makers. A wide variation in practices and beliefs emerged for communicating and engaging with policy makers. The study authors concluded that this may reflect the absence of several related but key supports for researchers regarding policy communication. Specifically, the authors discussed the lack of consensus on a common terminology or set of best practices or guidelines for communicating with policy makers, the lack of systematically designed training or mentorship, and the limited evidence on how research gets used in policymaking.

To further the PRIWG goal of identifying suggestions for improving how researchers engage with policy makers to get research into policy pathways, PRIWG aims to secure support to use these findings to inform the development of a larger, online survey of the field at large about knowledge, practices, experiences, and challenges of communicating and engaging with policy makers. In addition, PRIWG has established a transdisciplinary subgroup that is working to identify peer-reviewed articles that provide insights on how nutrition and obesity policy research gets used by elected officials in the United States. We also plan to identify any factors that influence the role of policy makers in helping to shape the research agenda and that could strengthen the design of policy-relevant studies. That is, we are exploring as best we can, with existing literature specific to nutrition and obesity policy research, the bidirectional researcher–policy maker relationship. Preliminary findings from the review of articles indicate that few researchers and funding sources are tackling this area of research; even fewer have a particular focus on nutrition and obesity policy research issues and opportunities.

As of 2014, PRIWG is sharing evidence gathered and exploring possible collaborative projects with the National Collaborative on Childhood Obesity Research (NCCOR) Get Research Used Workgroup (GRU). NCCOR brings together 4 of the nation’s leading funders —the CDC, National Institutes of Health (NIH), Robert Wood Johnson Foundation, and US Department of Agriculture — to improve the efficiency, effectiveness, and application of childhood obesity research and to halt — and reverse — childhood obesity (http://nccor.org/about/index). NCCOR focuses on efforts that have the potential to benefit children, teens, their families, and the communities in which they live. A special emphasis is placed on the populations and communities in which obesity rates are highest and rising the fastest. GRU grew out of recommendations put forth by the NCCOR External Scientific Panel, which serves as a liaison between NCCOR and the extramural community, informing NCCOR on new science and ideas and on connections to extramural research, practice, and policy (http://nccor.org/about/nesp). NCCOR External Scientific Panel members in 2012 and 2013 acknowledged that most research around childhood obesity probably does not get used and that childhood obesity researchers need to increase their capacity and skills to ensure their work reaches and resonates with key audiences. The panel recognized there were few incentives for researchers to actively work toward the translation and dissemination of their research and even fewer resources to help them. Therefore, GRU aims to empower researchers to translate and actively disseminate their results and findings and is considering, where needed, to develop resources designed to build researchers’ skills around policy research translation.

PRIWG’s next steps will be built on the notion that effective engagement with policy makers is not simply communicating and disseminating the end result of a research study but an active and bidirectional process from study conception to dissemination. Cultivating these relationships will require sensitivity to any institutional or funding source anti-lobbying guidance that may encourage translation and dissemination but prohibit advocacy activities (one federal example is US Office of Management and Budget Circular A-122). Moreover, developing strategic and systematic approaches to enhance how researchers engage with policy makers to get research considered and prioritized during the policymaking process will most likely require collaboration, tweaking, and tailoring to fit the particular nuances of the research and the needs of the researchers, policy makers, constituents, and stakeholders. Consideration must also be given to the role of intermediaries and disseminating organizations, such as advocacy groups, for facilitating the uptake of research into policy pathways (7).

Engaging with a policy maker or disseminating organization in any jurisdiction requires building trust and takes time — a precious commodity, especially for junior researchers. Like the NCCOR External Scientific Panel, we appreciate that there is limited and inconsistent preprofessional training or continued professional training for academic researchers on how to effectively engage with policy makers and few incentives encouraging researchers to do so. Brownson and colleagues have identified numerous factors hindering the translation of scientific evidence into public policy such as differences in decision making and persuasion among researchers and policy makers, ambiguous findings, and the need to balance objectivity and advocacy (8,9). At the same time, Brownson and colleagues have put forth solution-oriented suggestions for more effectively communicating findings to policy makers, including publishing scientific articles particularly focused on policy-relevant issues, reporting characteristics related to implementation and external validity, and taking additional actions across the advocacy continuum such as developing short policy summaries. Another suggestion put forth by Brownson and colleagues focuses on improved training and capacity building of students and professionals. Possible informal and formal learning strategies would first cover how to design and conduct rigorous quantitative and qualitative policy-relevant research and then how to get this research used. Other didactic and practicum educational offerings could focus on how to engage in partnerships with disseminating organizations and policy makers and communicate concisely and in straightforward language in both written and oral policy-relevant modes, mediums, and communication channels. Equally important, researchers could benefit from training and capacity building on how to best identify in their domain and for their relevant jurisdiction(s) the most effective way in real time to frame nutrition and obesity research that resonates with their relevant policy maker(s) (10).

Before training and capacity building along the pipeline of training from graduate school to senior investigators can be employed most effectively, a need exists to stimulate big picture and systematic thinking around ways to elevate the impact of nutrition and obesity policy research. Informed by and built on our formative transdisciplinary activities, PRIWG aims to work further on examining how to most effectively infuse policy research and evaluation work into policy pathways, convene thought leaders on this subject, canvass researcher and policy maker needs, and collect stories of both success and challenge. PRIWG also plans to draw on domains such as tobacco control and other disciplines such as public administration that have made substantial progress in promoting information dissemination and evidence uptake. Thus, PRIWG has developed a collaborative group and approach to move forward on its ultimate goal of identifying how best to elevate the impact of research and evaluation into policy pathways to make and improve on policies that support access to healthier food and beverage options and promote healthier food choices.
